# May the Children Help?

**Published:** 1893-06-03

**Authors:** 


### MAY THE CHILDREN HELP?

It has been suggested that the children of the richer classes at home, and the thousands of children who attend Sunday schools might be pleased with the opportunity of contributing their share to the church collections of the Hospital Sunday Fund. Those who have not specially thought of the subject are inclined to ask why the children's poor little minds should be worried about hospital collections, and why their small pence should be diverted from chocolates and toys into the channel of hospital support? Our experience of children is that their minds are by no means worried when they are applied to on the subject of giving, but on the contrary, they are usually very proud to be invited to share the responsibilities of their elders. There are, indeed, some persons who say that the hospitals are quite out of date, and show an extraordinary lack of modern enterprise in not having enlisted the sympathy and help of the children long ago. We are inclined to believe there is some truth in this. The enterprising total abstainer has been much wiser in his day, and has made an effort to include the whole rising generation in his Bands of Hope and Temperance Guilds. So, too, have the Missionary Societies seen their account in enlisting the sympathies of the young on behalf of the heathen children in distant lands. But are there no children's hospitals, and no children's cots in general hospitals, where suffering and helpless little one lie; little ones whom their happier and more prosperous fellow-children would gladly smile or cry with, or still more gladly help to happiness and recovery by contributing out of a generous self-denial a few spare pence, or even spare shillings or half-crowns? The children of to-day will be grown men and women before we elders know where we are, and they will be likely to be much more generous and sympathetic towards hospitals in middle and old age if they have been habitually generous towards them when young. The object of the supporters of voluntary hospitals is to persuade the whole population to voluntarily tax itself according to its means for the help of the sick poor. In no other way can this desirable and statesmanlike object be so widely accomplished as by the systematic training of children in hospital support.

